# QuEChERS-在线凝胶渗透色谱-气相色谱-串联质谱法高通量筛查动物源性食品中的多农药残留

**DOI:** 10.3724/SP.J.1123.2022.10010

**Published:** 2023-07-08

**Authors:** Jie LI, Xiang JU, Yanli WANG, Qiyan TIAN, Xiuqing LIANG, Haixia LI, Yanming LIU

**Affiliations:** 山东省食品药品检验研究院,国家市场监管重点实验室(肉及肉制品监管技术),山东省特殊医学用途配方食品质量控制工程技术研究中心,山东省食品药品安全检测工程技术中心,山东济南250101; Shandong Institute for Food and Drug Control, Key Laboratory of Supervising Technology for Meat and Meat Products for State Market Regulation, Shandong Research Center of Engineering and Technology for Quality Control of Food for Special Medical Purposes, Shandong Research Center of Engineering and Technology for Safety Inspection of Food and Drug, Jinan 250101, China

**Keywords:** QuEChERS, 在线凝胶渗透色谱, 气相色谱-串联质谱, 农药残留, 动物源性食品, QuEChERS, online gel permeation chromatography, gas chromatography-tandem mass spectrometry (GC-MS/MS), pesticide residues, animal-derived foods

## Abstract

将QuEChERS技术与在线凝胶渗透色谱-气相色谱-串联质谱(GPC-GC-MS/MS)技术结合,建立了动物源性食品中196种农药残留的高通量检测方法。样品经乙腈提取、QuEChERS结合在线GPC净化后,进行GC-MS/MS检测,多反应监测模式(MRM)分析,外标法定量。方法优化了提取溶剂及净化剂类型,实现了目标物的高效提取和基质的有效去除;考察了在线GPC对样品溶液的净化作用。通过研究不同馏分接收时间段内目标物的回收率和基质效应,得出最佳馏分接收时间,实现目标物的有效导入和基质的高效去除,并对QuEChERS-GPC联用技术的优势进行了评价。该方法研究了196种农药的基质效应,其中10种农药表现为中等强度基质效应,4种农药为强基质效应。本研究采用基质匹配标准溶液进行定量,结果表明,196种农药在0.005~0.2 mg/L范围内线性关系良好,相关系数均>0.996,方法的检出限为0.002 mg/kg,定量限为0.005 mg/kg。向猪肉、牛肉、猪肝3种不同脂肪含量基质的空白样品中添加标准溶液,196种农药在3个加标水平下(0.01、0.05、0.20 mg/kg)的平均回收率为65.3%~126.2%,精密度为0.7%~5.7%。本方法快速、准确、灵敏,适用于动物源性食品中多农药残留的高通量筛查与检测。

## Abstract (en)

Improvements in living standards have led to an increase in the consumption of animal-derived foods. Pesticides may be used illegally during animal breeding as well as meat production and processing for pest control and preservation. Pesticides applied to crops may also be enriched in animal tissues through the food chain, thereby increasing the risk of pesticide residue accumulation in muscles and visceral tissues and endangering human health. China has stipulated maximum residue limits for pesticide residues in livestock and poultry meat and their viscera. Many other major developed countries and organizations, including the European Union, Codex Alimentarius Commission, and Japan, have also set maximum residue limits for these residues (0.005-10, 0.004-10, and 0.001-10 mg/kg, respectively). Research on pretreatment technologies for pesticide residue detection in plant-derived foods is widely available, but insufficient attention has been paid to animal-derived foods. Thus, high-throughput detection technologies for pesticide residues in animal-derived foods are limited. The impurities that can interfere with the detection process for plant-derived foods mainly include organic acids, polar pigments, and other small molecular compounds; by contrast, the matrix of animal-derived foods is much more complex. Macromolecular proteins, fats, small molecular amino acids, organic acids, and phospholipids can interfere with the detection of pesticide residues in animal-derived foods. Thus, selecting the appropriate pretreatment and purification technology is of great importance. In this study, the QuEChERS technique was combined with online gel permeation chromatography-gas chromatography-tandem mass spectrometry (GPC-GC-MS/MS) to determine 196 pesticide residues in animal-derived foods. The samples were extracted with acetonitrile, purified using the QuEChERS technique coupled with online GPC, detected by GC-MS/MS, determined in multiple reaction monitoring mode (MRM), and quantified using the external standard method. The effects of the extraction solvent and purification agent type on the extraction efficiency and matrix removal of the method were optimized. The purification effect of online GPC on the sample solution was investigated. The optimal distillate receiving time was obtained by studying the recoveries of the target substances and matrix effects over different distillate receiving periods to achieve the effective introduction of target substances and efficient matrix removal. Further, the advantages of the QuEChERS technique combined with online GPC were evaluated. The matrix effects of 196 pesticides were assessed; ten pesticide residues showed moderate matrix effects, while four pesticide residues showed strong matrix effects. A matrix-matched standard solution was used for quantification. The 196 pesticides showed good linearity in the range of 0.005-0.2 mg/L, with correlation coefficients greater than 0.996. The limits of detection and quantification were 0.002 and 0.005 mg/kg, respectively. The recoveries of 196 pesticides at spiked levels of 0.01, 0.05, and 0.20 mg/kg were 65.3%-126.2%, with relative standard deviations (RSDs) of 0.7%-5.7%. The proposed method is rapid, accurate, and sensitive; thus, it is suitable for the high-throughput screening and detection of multiple pesticide residues in animal-derived foods.

随着人民生活水平的提高,动物源性食品在饮食中的比重日益增大,但在动物养殖、肉类生产加工等环节为驱虫保鲜可能会违法使用农药;此外,作用在农作物上的农药也可能通过食物链的方式富集在动物组织中,使得肌肉、内脏组织中农药残留的风险增大,给人们的身体健康带来一定的危害^[1,2]^。目前欧盟、国际食品法典委员会(CAC)、日本等主要发达国家及组织分别规定了畜禽肉及其内脏中500、287、159种农药的最大残留限量(MRL),限量要求分别为0.005~10 mg/kg、0.004~10 mg/kg、0.001~10 mg/kg。我国自2005年发布《食品安全国家标准 食品中农药最大残留限量》至2021年,对畜禽肉及其内脏中农药残留限量的规定由3种增加到133种,MRL要求为0.005~3 mg/kg^[3,4]^,说明我国也越来越重视动物源性食品中的农药残留问题。但在规定的133种农药中有86种未指定检验方法,缺少配套的检验标准,需要开发相关的检测技术。

食品中农药残留的高通量检测方法较多,主要有气相色谱法(GC)、气相色谱-质谱法(GC-MS)、气相色谱-串联质谱法(GC-MS/MS)、液相色谱法(LC)、液相色谱-质谱法(LC-MS)、液相色谱-串联质谱法(LC-MS/MS)及高分辨质谱法(HRMS)^[5⇓⇓⇓⇓⇓⇓⇓⇓-14]^等。但目前农药残留检测的前处理技术更关注于植物源性食品,对于动物源性食品研究较少。对于植物源性食品,干扰检测的杂质更多是有机酸、极性色素等小分子化合物,而动物源性食品基质复杂,农药的检测会同时受到大分子蛋白质、脂肪、小分子氨基酸、有机酸、磷脂等的共同干扰^[15]^,因此选择合适的样品前处理净化技术尤为关键。目前去除脂肪、蛋白质、色素等大分子杂质的前处理方法多采用凝胶渗透色谱(GPC)净化和冷冻除脂等,现有的国家标准及文献报道普遍采取离线GPC净化的方式对样品提取液进行净化^[16⇓-18]^,但离线的GPC溶剂消耗量大,耗时长,不适用于批量样品的检测;小分子有机酸、极性色素和糖类等杂质多采用固相萃取或QuEChERS方法进行去除^[2,19⇓⇓-22]^。李桂琴等^[2]^利用磁性固相萃取净化技术测定肉类及肉类产品中的有机氯和菊酯类农药;李曼曼等^[19]^利用QuEChERS方法测定畜禽肉中的17种有机氯农药。现有报道的动物源性食品中农药残留的前处理技术较多,但是综合考虑其同时去除大、小分子杂质能力的研究较少。

目前针对动物源性食品中农药残留的检测虽有一些文献报道,但检测的目标物多为单个或者单种类别(有机磷、有机氯或菊酯类等)的化合物,高通量检测的方法较少。本文选取国内外关注较多的196种农药作为目标物,采用QuEChERS结合在线GPC净化技术,对动物源性食品中的大、小分子杂质同时进行净化,通过优化样品前处理技术及色谱、质谱分析条件,建立了同时测定肌肉组织(牛肉、猪肉、羊肉、鸡肉)、内脏组织(猪肝、猪肾、猪肺、牛肝)等动物源性食品中196种农药残留的高通量气相色谱-串联质谱检测方法。该方法净化效果好,检测通量高,适用基质范围广,可快速、准确地测定动物源性食品中的农药残留。

## 1 实验部分

### 1.1 仪器、试剂与材料

QP2030-TQ8040在线凝胶渗透色谱-气相色谱-串联质谱联用仪(日本岛津公司),配有电子轰击电离源;MS204S电子天平(瑞士梅特勒-托利多集团);Milli-Q超纯水仪(美国Millipore公司);3-18K高速冷冻离心机(德国SIGMA公司);涡旋混合器(德国IKA公司)。

乙酸乙酯、乙腈、丙酮(高效液相色谱级,德国Merck公司);聚苯乙烯凝胶(Bio-Beads S-X3200-400目,美国Bio-Rad公司);无水硫酸钠、氯化钠(分析纯,国药集团化学试剂有限公司);*N*-丙基乙二胺(PSA)(天津博纳艾杰尔科技有限公司);十八烷基硅烷(C18)(上海安谱科技股份有限公司);QuEChERS净化管、HLB固相萃取柱、C18固相萃取柱(美国Waters公司);氨基柱(NH_2_)、弗罗里硅土柱(中国美正检测公司);EMR-lipid净化剂、惰性石英管(5 m×0.53 mm,美国Agilent公司);196种农药混合标准溶液具体名称见表1,质量浓度均为10 μg/mL(北京振翔公司)。

**表1:** 196种农药的保留时间、定量及定性离子对、碰撞能量、线性方程、相关系数(*r*)及定量限

No.	Pesticide	Retention time/min	Quantitative and qualitative ion pairs (*m/z*)	CEs/eV	Linear equation	*r*	LOQ/ (mg/kg)
1	ethiolate (硫草敌)	5.595	100.00>72.10, 161.00>100.10	6, 9	*y*=3.58×10^5^*x*-68.1	0.9997	0.005
2	dichlorvos (敌敌畏)	6.049	109.00>79.00, 185.00>93.00	8, 14	*y*=3.33×10^5^*x*+3.13×10^2^	0.9997	0.005
3	dichlorobenzonitrile (敌草腈)	7.058	170.90>136.00, 170.90>100.00	14, 24	*y*=4.33×10^5^*x*-1.34×10^2^	0.9996	0.005
4	biphenyl (联苯)	7.392	154.10>128.10, 154.10>115.10	22, 24	*y*=1.37×10^5^*x*-69.4	0.9995	0.005
5	mevinphos (速灭磷)	7.865	127.00>109.00, 192.00>127.00	12, 12	*y*=5.39×10^5^*x*-1.92×10^3^	0.9995	0.005
6	etridiazole (土菌灵)	8.154	210.90>182.90, 182.90>139.90	10, 18	*y*=2.26×10^5^*x*-8.25×10^2^	0.9993	0.005
7	methacrifos (虫螨畏)	8.641	208.00>180.00, 240.00>208.00	8, 4	*y*=2.95×10^5^*x*-8.06×10^2^	0.9995	0.005
8	chloroneb (氯苯甲醚)	8.727	206.00>141.00, 193.00>113.00	20, 18	*y*=1.06×10^5^*x*-3.24×10^2^	0.9993	0.005
9	molinate (禾草敌)	9.026	187.10>126.10, 126.10>55.00	6, 14	*y*=2.57×10^5^*x*-9.60×10^2^	0.9995	0.005
10	isoprocarb (异丙威)	9.040	136.00>121.00, 121.00>77.00	10, 22	*y*=1.07×10^5^*x*-3.87×10^3^	0.9993	0.005
11	thionazin (虫线磷)	9.773	143.00>79.10, 175.00>79.10	12, 12	*y*=1.78×10^5^*x*-6.00×10^2^	0.9995	0.005
12	tecnazene (四氯硝基苯)	9.786	260.90>202.90, 202.90>142.90	14, 22	*y*=9.18×10^4^*x*-3.76×10^2^	0.9992	0.005
13	propoxur (残杀威)	9.833	152.10>110.10, 110.10>64.00	8, 18	*y*=5.86×10^5^*x*-2.14×10^3^	0.9992	0.005
14	diphenylamine (二苯胺)	9.962	169.10>66.00, 167.10>139.10	24, 28	*y*=1.73×10^5^*x*-4.69×10^2^	0.9991	0.005
15	ethoprophos (灭线磷)	10.067	200.00>158.00, 158.00>97.00	6, 18	*y*=2.66×10^5^*x*-1.09×10^3^	0.9991	0.005
16	cycloate (环草敌)	10.068	154.10>83.10, 83.10>55.00	10, 10	*y*=4.43×10^5^*x*-6.78×10^2^	0.9995	0.005
17	chlorpropham (氯苯胺灵)	10.246	127.10>65.00, 213.10>171.10	22, 6	*y*=2.37×10^5^*x*-6.12×10^2^	0.9997	0.005
18	ethalfluralin (乙丁烯氟灵)	10.329	276.00>202.00, 316.10>276.00	18, 10	*y*=9.94×10^4^*x*-3.30×10^2^	0.9996	0.005
19	deethylatrazine (脱乙基莠去津)	10.378	187.00>172.10, 172.00>69.10	6, 18	*y*=1.56×10^5^*x*-6.00×10^2^	0.9994	0.005
20	dicrotofos (百治磷)	10.484	127.10>109.00, 127.10>95.00	12, 18	*y*=5.12×10^5^*x*-1.54×10^3^	0.9997	0.005
21	benfluralin (氟草胺)	10.548	292.10>264.00, 292.10>160.00	8, 22	*y*=2.30×10^5^*x*-8.38×10^2^	0.9997	0.005
22	monocrotophos (久效磷)	10.483	127.10>109.00, 127.10>95.00	12, 16	*y*=5.15×10^5^*x*-2.24×10^3^	0.9998	0.005
23	sulfotep (治螟磷)	10.618	322.00>202.00, 322.00>174.00	10, 18	*y*=1.58×10^5^*x*-5.09×10^2^	0.9998	0.005
24	phorate (甲拌磷)	10.717	260.00>75.00, 231.00>129.00	12, 16	*y*=1.12×10^5^*x*-3.68×10^2^	0.9998	0.005
25	*α*-BHC (*α*-六六六)	10.819	180.90>144.90, 218.90>182.90	16, 8	*y*=3.56×10^5^*x*-1.10×10^2^	0.9996	0.005
26	hexachlorobenzene (六氯苯)	10.972	283.80>248.80, 283.80>213.80	24, 28	*y*=2.47×10^5^*x*-8.00×10^2^	0.9994	0.005
27	dicloran (氯硝胺)	11.003	206.00>176.00, 176.00>148.00	10, 12	*y*=1.66×10^5^*x*-4.52×10^2^	0.9997	0.005
28	atratone (阿特拉通)	11.070	211.00>169.20, 211.00>154.10	6, 15	*y*=1.79×10^5^*x*-5.57×10^2^	0.9999	0.005
29	dimethoate (乐果)	11.117	125.00>47.00, 125.00>79.00	14, 8	*y*=1.00×10^5^*x*-3.83×10^2^	0.9998	0.005
30	simazine (西玛津)	11.191	201.10>173.10, 201.10>186.10	6, 6	*y*=1.84×10^5^*x*-7.56×10^2^	0.9998	0.005
31	carbofuran (克百威)	11.226	164.10>149.10, 149.10>121.10	8, 10	*y*=3.35×10^5^*x*-1.16×10^2^	0.9998	0.005
32	atrazine (莠去津)	11.296	215.10>58.00, 215.10>173.10	14, 6	*y*=9.38×10^4^*x*-4.01×10^2^	0.9998	0.005
33	monolinuron (绿谷隆)	11.311	214.00>61.00, 126.00>99.00	10, 15	*y*=1.18×10^5^*x*-4.25×10^2^	0.9998	0.005
34	propazine (扑灭津)	11.379	229.10>58.00, 229.10>187.10	14, 6	*y*=1.04×10^5^*x*-3.30×10^2^	0.9999	0.005
35	clomazone (异噁草酮)	11.349	204.10>107.00, 204.10>78.00	20, 26	*y*=3.07×10^5^*x*-1.26×10^3^	0.9993	0.005
36	*β*-BHC (*β*-六六六)	11.406	180.90>144.90, 218.90>182.90	16, 8	*y*=5.67×10^5^*x*-1.93×10^3^	0.9991	0.005
37	*γ*-BHC (*γ*-六六六)	11.491	180.90>144.90, 218.90>182.90	16, 8	*y*=5.66×10^5^*x*-1.95×10^2^	0.9991	0.005
38	propetamphos (胺丙畏)	11.592	138.00>110.00, 138.00>64.00	10, 15	*y*=4.63×10^5^*x*-1.91×10^3^	0.9998	0.005
39	terbufos (特丁硫磷)	11.583	231.00>128.90, 231.00>174.90	26, 14	*y*=3.65×10^5^*x*-1.42×10^3^	0.9993	0.005
40	pentachloronitrobenzene (五氯硝基苯)	11.588	264.80>236.80, 294.80>236.80	10, 16	*y*=7.28×10^4^*x*-2.02×10^2^	0.9993	0.005
41	profluralin (环丙氟灵)	11.628	318.00>199.10, 318.00>55.10	18, 18	*y*=6.30×10^4^*x*-1.88×10^2^	0.9993	0.005
42	pronamide (炔苯酰草胺)	11.653	172.90>144.90, 172.90>109.00	16, 26	*y*=7.08×10^5^*x*-3.01×10^3^	0.9998	0.005
43	pyrimethanil (嘧霉胺)	11.761	198.10>183.10, 198.10>118.10	14, 28	*y*=2.17×10^5^*x*-8.98×10^2^	0.9993	0.005
44	diazinon (二嗪磷)	11.823	304.10>179.10, 179.10>137.10	10, 18	*y*=1.43×10^5^*x*-5.50×10^2^	0.9999	0.005
45	phosphamidon (磷胺)	11.861	127.10>109.10, 127.10>95.10	12, 18	*y*=1.18×10^5^*x*-3.33×10^2^	0.9993	0.005
		12.788					
46	*δ*-BHC (*δ*-六六六)	12.006	180.90>144.90, 218.90>182.90	16, 8	*y*=2.42×10^5^*x*-8.68×10^2^	0.9998	0.005
47	isazofos (氯唑磷)	12.086	257.00>162.00, 257.00>119.00	8, 18	*y*=9.33×10^4^*x*-3.79×10^2^	0.9990	0.005
48	triallate (野麦畏)	12.090	268.10>184.00, 270.10>186.00	20, 20	*y*=2.04×10^5^*x*-8.24×10^2^	0.9994	0.005
49	etrimfos (乙嘧硫磷)	12.126	181.10>153.10, 292.10>181.10	10, 8	*y*=4.59×10^5^*x*-1.87×10^3^	0.9991	0.005
50	tebupirimfos (丁基嘧啶磷)	12.219	261.10>137.10, 318.10>152.10	18, 14	*y*=1.60×10^5^*x*-4.65×10^2^	0.9991	0.005
51	iprobenfos (异稻瘟净)	12.251	204.00>91.00, 204.00>122.00	8, 12	*y*=5.91×10^5^*x*-2.54×10^3^	0.9994	0.005
52	pirimicarb (抗蚜威)	12.344	238.10>166.10, 166.10>55.00	12, 20	*y*=3.48×10^5^*x*-1.13×10^3^	0.9996	0.005
53	formothion (安硫磷)	12.355	170.00>93.00, 224.00>125.00	8, 18	*y*=1.45×10^5^*x*-6.94×10^2^	0.9995	0.005
54	pentachloroaniline (五氯苯胺)	12.427	262.80>191.90, 264.80>193.90	21, 21	*y*=1.63×10^5^*x*-6.43×10^2^	0.9994	0.005
55	desmetryn (敌草净)	12.515	213.00>171.10, 213.00>58.10	6, 18	*y*=1.98×10^5^*x*-9.34×10^2^	0.9992	0.005
56	dichlofenthion (除线磷)	12.577	279.00>222.90, 222.90>204.90	14, 14	*y*=4.64×10^5^*x*-1.36×10^3^	0.9994	0.005
57	propanil (敌稗)	12.696	217.00>161.00, 160.90>99.00	10, 24	*y*=2.15×10^5^*x*-8.69×10^2^	0.9998	0.005
58	metribuzin (嗪草酮)	12.632	198.10>82.00, 198.10>110.10	14, 10	*y*=1.02×10^5^*x*-4.59×10^2^	0.9998	0.005
59	acetochlor (乙草胺)	12.723	174.10>146.10, 223.10>132.10	12, 22	*y*=1.76×10^5^*x*-6.96×10^2^	0.9998	0.005
60	vinclozolin (乙烯菌核利)	12.762	197.00>124.00, 197.97>145.00	10, 10	*y*=8.30×10^4^*x*-3.59×10^2^	0.9995	0.005
61	chlorpyrifos (甲基毒死蜱)	12.771	285.90>93.00, 287.90>93.00	22, 22	*y*=1.35×10^5^*x*-5.63×10^2^	0.9998	0.005
62	parathion (甲基对硫磷)	12.776	263.00>109.00, 125.00>47.00	14, 12	*y*=2.36×10^5^*x*-1.00×10^3^	0.9995	0.005
63	malaoxon (马拉氧磷)	12.788	126.90>99.00, 268.00>126.90	10, 10	*y*=4.12×10^5^*x*-1.53×10^3^	0.9990	0.005
64	tolclofos (甲基立枯磷)	12.863	264.90>249.90, 264.90>93.00	14, 24	*y*=3.84×10^5^*x*-1.24×10^3^	0.9996	0.005
65	alachlor (甲草胺)	12.917	188.10>160.10, 188.10>132.10	10, 18	*y*=2.71×10^5^*x*-4.46×10^2^	0.9995	0.005
66	ametryn (莠灭净)	12.932	227.10>185.10, 227.10>58.00	6, 14	*y*=1.56×10^5^*x*-6.56×10^2^	0.9992	0.005
67	prometryn (扑草净)	12.998	226.10>184.10, 241.20>184.10	10, 12	*y*=1.81×10^5^*x*-8.15×10^2^	0.9991	0.005
68	paraoxon (对氧磷)	13.025	109.00>91.00, 148.90>119.00	6, 5	*y*=9.74×10^4^*x*-4.89×10^2^	0.9994	0.005
69	metalaxyl (甲霜灵)	13.020	249.20>190.10, 206.10>132.10	8, 20	*y*=1.16×10^5^*x*-4.87×10^2^	0.9998	0.005
70	ronnel (皮蝇磷)	13.053	284.90>269.90, 286.90>271.90	16, 18	*y*=3.06×10^5^*x*-1.26×10^3^	0.9995	0.005
71	terbutryn (去草净)	13.251	241.20>185.10, 241.20>170.10	6, 14	*y*=3.46×10^5^*x*-1.13×10^3^	0.9991	0.005
72	fenitrothion (杀螟硫磷)	13.314	277.00>260.00, 277.00>109.10	6, 14	*y*=2.18×10^5^*x*-9.51×10^2^	0.9996	0.005
73	pirimiphos (甲基嘧啶磷)	13.338	290.10>125.00, 290.10>233.10	22, 12	*y*=1.59×10^5^*x*-4.60×10^2^	0.9993	0.005
74	ethofumesate (灭草松)	13.364	207.10>161.10, 207.10>137.10	8, 12	*y*=2.53×10^5^*x*-1.02×10^3^	0.9990	0.005
75	phoratesulfoxide (甲拌磷亚砜)	13.630	153.00>97.00, 199.00>171.10	12, 6	*y*=4.68×10^5^*x*+1.46×10^3^	0.9996	0.005
76	dipropetryn (异丙净)	13.487	255.00>222.20, 255.00>180.20	9, 18	*y*=2.37×10^5^*x*-6.85×10^2^	0.9991	0.005
77	malathion (马拉硫磷)	13.509	173.10>99.00, 173.10>127.00	14, 6	*y*=4.12×10^5^*x*-1.75×10^3^	0.9995	0.005
78	thiobencarb (禾草丹)	13.518	100.00>72.00, 125.00>89.00	5, 18	*y*=8.08×10^5^*x*-3.64×10^3^	0.9993	0.005
79	aldrin (艾氏剂)	13.593	262.90>191.00, 262.90>193.00	34, 28	*y*=5.78×10^4^*x*-2.23×10^2^	0.9998	0.005
80	phoratesulfone (甲拌磷砜)	13.631	153.00>97.00, 153.00>125.00	12, 6	*y*=4.67×10^5^*x*-8.04×10^2^	0.9997	0.005
81	metolachlor (异丙甲草胺)	13.635	162.10>133.10, 238.10>162.10	16, 12	*y*=1.03×10^6^*x*-2.15×10^3^	0.9994	0.005
82	fenthion (倍硫磷)	13.683	278.00>109.00, 278.00>169.00	20, 14	*y*=2.99×10^5^*x*-7.89×10^2^	0.9994	0.005
83	chlorpyrifos (毒死蜱)	13.720	196.90>168.90, 313.90>257.90	14, 14	*y*=2.09×10^5^*x*-4.66×10^2^	0.9995	0.005
84	parathion (对硫磷)	13.742	291.10>109.00, 139.00>109.00	14, 8	*y*=1.52×10^5^*x*-4.95×10^2^	0.9994	0.005
85	triadimefon (三唑酮)	13.785	208.10>181.00, 208.10>111.00	10, 22	*y*=2.33×10^5^*x*-1.03×10^3^	0.9991	0.005
86	isofenphosoxon (氧异柳磷)	13.855	229.10>201.00, 201.00>121.00	10, 20	*y*=1.14×10^6^*x*-4.68×10^3^	0.9998	0.005
87	isocarbophos (水胺硫磷)	13.866	289.10>136.00, 230.00>212.00	14, 10	*y*=8.83×10^4^*x*-3.02×10^2^	0.9998	0.005
88	tetraconazole (氟醚唑)	13.888	336.00>204.00, 336.00>218.00	28, 14	*y*=6.57×10^4^*x*-1.94×10^2^	0.9997	0.005
89	trichloronat (毒壤膦)	13.969	297.00>269.00, 299.00>271.00	15, 15	*y*=3.83×10^5^*x*-1.07×10^3^	0.9994	0.005
90	fosthiazate (噻唑膦)	14.063	195.00>103.00, 195.00>60.00	10, 22	*y*=1.68×10^5^*x*-5.53×10^2^	0.9998	0.005
		14.111					
91	bromophos (溴硫磷)	14.065	330.90>315.90, 328.90>313.90	14, 18	*y*=1.53×10^5^*x*-3.98×10^2^	0.9992	0.005
92	pirimiphos (乙基虫螨磷)	14.136	304.00>168.00, 318.00>166.00	10, 15	*y*=1.76×10^5^*x*-7.72×10^2^	0.9995	0.005
93	isofenphos (甲基异柳磷)	14.228	199.00>121.00, 241.10>121.10	14, 22	*y*=7.62×10^5^*x*-3.29×10^3^	0.9992	0.005
94	terbufossulfone (特丁硫磷砜)	14.376	153.00>97.00, 199.00>97.00	21, 21	*y*=2.88×10^5^*x*-1.08×10^3^	0.9998	0.005
95	(*E*)-chlorfenvinphos ((*E*)-毒虫畏)	14.515	323.00>267.00, 267.00>159.00	16, 18	*y*=2.53×10^5^*x*-1.02×10^3^	0.9990	0.005
96	pendimethalin (二甲戊灵)	14.343	252.10>162.10, 252.10>191.10	10, 8	*y*=1.42×10^5^*x*-5.22×10^2^	0.9995	0.005
97	penconazole (戊菌唑)	14.378	248.10>157.10, 159.10>123.10	26, 22	*y*=3.33×10^5^*x*-1.34×10^3^	0.9991	0.005
98	mephosfolan (地胺磷)	14.527	196.00>140.00, 196.00>168.00	12, 6	*y*=3.72×10^5^*x*-1.68×10^3^	0.9995	0.005
99	isofenphos (异柳磷)	14.517	213.00>121.00, 213.00>185.00	15, 6	*y*=5.79×10^5^*x*-2.37×10^3^	0.9991	0.005
100	(*Z*)-chlorfenvinphos ((*Z*)-毒虫畏)	14.515	323.00>267.00, 267.00>159.00	16, 18	*y*=2.53×10^5^*x*-9.01×10^2^	0.9991	0.005
101	fipronil (氟虫腈)	14.558	366.90>212.90, 368.90>214.90	30, 30	*y*=1.41×10^5^*x*-3.41×10^2^	0.9992	0.005
102	beflubutamid (氟丁酰草胺)	14.562	176.00>91.10, 221.00>193.00	15, 12	*y*=4.43×10^5^*x*-2.00×10^3^	0.9993	0.005
103	procymidone (腐霉利)	14.692	283.00>96.00, 285.00>96.00	10, 10	*y*=1.68×10^5^*x*-4.87×10^2^	0.9995	0.005
104	chlordane (氯丹-反式)	14.829	374.80>265.90, 372.80>263.90	26, 28	*y*=1.29×10^5^*x*-2.95×10^2^	0.9992	0.005
105	methidathion (杀扑磷)	14.850	145.00>85.00, 145.00>58.00	8, 14	*y*=1.05×10^6^*x*-4.23×10^2^	0.9998	0.005
106	bromophos (乙基溴硫磷)	14.884	358.90>302.90, 302.90>284.90	16, 18	*y*=2.66×10^5^*x*-9.60×10^2^	0.9993	0.005
107	tetrachlorvinphose (杀虫畏)	15.034	328.90>109.00, 330.90>109.00	20, 22	*y*=2.30×10^5^*x*-1.06×10^3^	0.9998	0.005
108	fenothiocarb (苯硫威)	14.947	160.10>72.00, 160.10>106.10	10, 12	*y*=4.77×10^5^*x*-1.99×10^3^	0.9996	0.005
109	*o*,*p*'-DDE (*o*,*p*'-滴滴伊)	14.917	246.00>176.00, 248.00>176.00	30, 28	*y*=4.84×10^5^*x*-1.60×10^3^	0.9991	0.005
110	paclobutrazol (多效唑)	14.948	236.10>125.00, 236.10>167.00	14, 10	*y*=4.29×10^5^*x*-1.60×10^3^	0.9998	0.005
111	mepanipyrim (嘧菌胺)	15.081	222.10>221.10, 223.10>222.10	6, 10	*y*=1.73×10^6^*x*-7.18×10^3^	0.9998	0.005
112	*α*-endosulfan (*α*-硫丹)	15.066	194.90>160.00, 194.90>125.00	8, 24	*y*=2.37×10^4^*x*+2.00	0.9995	0.005
113	butachlor (丁草胺)	15.100	176.10>147.10, 188.10>160.10	14, 12	*y*=2.01×10^5^*x*-6.12×10^2^	0.9994	0.005
114	ditalimfos (灭菌磷)	15.164	130.00>102.10, 148.00>130.10	10, 10	*y*=5.34×10^5^*x*-1.68×10^3^	0.9992	0.005
115	chlorfenson (杀螨酯)	15.251	175.00>111.00, 175.00>75.00	10, 10	*y*=9.40×10^5^*x*-4.04×10^3^	0.9998	0.005
116	bromfenvinfos (溴苯烯磷)	15.281	267.00>159.00, 269.00>161.00	15, 21	*y*=3.23×10^5^*x*-1.45×10^3^	0.9998	0.005
117	butamifos (抑草磷)	15.272	286.10>202.10, 200.10>65.00	14, 22	*y*=2.83×10^5^*x*-1.05×10^3^	0.9996	0.005
118	napropamide (萘丙酰草胺)	15.282	128.00>72.10, 100.00>72.00	6, 8	*y*=3.89×10^5^*x*-1.96×10^3^	0.9998	0.005
119	flutolanil (氟酰胺)	15.323	173.00>145.00, 173.00>95.00	14, 26	*y*=1.98×10^6^*x*-5.31×10^3^	0.9994	0.005
120	hexaconazole (己唑醇)	15.322	214.00>159.00, 214.00>172.00	20, 20	*y*=7.09×10^4^*x*-1.67×10^2^	0.9998	0.005
121	imazalil (抑霉唑)	15.392	215.00>173.00, 215.00>159.00	6, 6	*y*=1.88×10^5^*x*-8.51×10^2^	0.9991	0.005
122	prothiofos (丙硫磷)	15.383	266.90>238.90, 309.00>238.90	10, 14	*y*=2.65×10^5^*x*-5.55×10^2^	0.9996	0.005
123	isoprothiolane (稻瘟灵)	15.401	231.10>189.00, 290.10>118.00	10, 14	*y*=1.48×10^5^*x*-1.11×10^2^	0.9993	0.005
124	profenofos (丙溴磷)	15.441	338.90>268.90, 336.90>266.90	18, 14	*y*=1.34×10^5^*x*-4.72×10^2^	0.9998	0.005
125	tribufos (脱叶磷)	15.494	202.00>147.00, 202.00>113.00	6, 20	*y*=2.35×10^5^*x*-1.09×10^3^	0.9998	0.005
126	pretilachlor (丙草胺)	15.487	262.10>202.10, 238.10>162.10	10, 10	*y*=2.37×10^5^*x*-9.80×10^2^	0.9998	0.005
127	*p*,*p*'-DDE (*p*,*p*'-滴滴伊)	15.512	246.00>176.00, 317.90>248.00	30, 24	*y*=4.06×10^5^*x*-3.02×10^2^	0.9995	0.005
128	oxadiazon (噁草酮)	15.574	258.00>175.00, 302.00>175.00	8, 14	*y*=2.22×10^5^*x*-5.18×10^2^	0.9990	0.005
129	dieldrin (狄氏剂)	15.562	276.90>241.00, 262.90>193.00	8, 34	*y*=2.76×10^4^*x*-37.8	0.9994	0.005
130	myclobutanil (腈菌唑)	15.661	179.10>125.00, 179.10>152.00	14, 8	*y*=5.07×10^5^*x*-1.35×10^3^	0.9994	0.005
131	oxyfluorfen (乙氧氟草醚)	15.676	252.00>196.00, 361.00>300.00	22, 14	*y*=7.17×10^4^*x*-2.79×10^2^	0.9994	0.005
132	*o*,*p*'-DDD (*o*,*p*'-滴滴滴)	15.677	235.00>165.00, 237.00>165.00	24, 28	*y*=9.26×10^5^*x*-1.96×10^3^	0.9996	0.005
133	bupirimate (乙嘧酚磺酸酯)	15.756	273.10>108.10, 273.10>193.10	16, 8	*y*=1.49×10^5^*x*-3.39×10^2^	0.9994	0.005
134	kresoxim (醚菌酯)	15.769	116.00>89.00, 116.00>63.00	15, 30	*y*=5.85×10^5^*x*-1.24×10^2^	0.9993	0.005
135	isoxathion (异唑磷)	15.877	177.10>130.10, 177.10>116.10	10, 12	*y*=1.76×10^5^*x*-6.22×10^2^	0.9976	0.005
136	nitrofen (除草醚)	15.926	202.00>139.00, 282.90>253.00	24, 12	*y*=1.76×10^5^*x*-6.05×10^2^	0.9997	0.005
137	fluazifop (吡氟禾草灵)	15.956	282.00>91.10, 282.00>238.10	18, 18	*y*=1.96×10^5^*x*-5.44×10^2^	0.9992	0.005
138	endrin (异狄氏剂)	15.970	262.90>191.00, 244.90>173.00	30, 32	*y*=4.60×10^4^*x*-2.02×10^2^	0.9997	0.005
139	chlorobenzilate (乙酯杀螨醇)	16.113	139.00>111.00, 251.00>139.00	16, 14	*y*=8.56×10^5^*x*-2.47×10^3^	0.9994	0.005
140	*β*-endosulfan (*β*-硫丹)	16.154	194.90>160.00, 194.90>125.00	8, 24	*y*=3.15×10^4^*x*-42.3	0.9993	0.005
141	fensulfothion (丰索磷)	16.218	291.80>156.00, 291.80>108.80	15, 15	*y*=1.50×10^5^*x*-6.89×10^2^	0.9995	0.005
142	chlorthiophos (虫螨磷)	16.043	324.90>268.90, 268.90>205.00	10, 5	*y*=1.34×10^4^*x*-75.3	0.9995	0.005
		16.441					
143	fenthionsulfoxide (倍硫磷亚砜)	16.235	278.00>108.90, 278.00>125.10	18, 24	*y*=1.48×10^4^*x*+5.53×10^2^	0.9965	0.005
144	diniconazole (烯唑醇)	16.247	268.00>232.00, 270.00>234.00	12, 10	*y*=2.96×10^5^*x*-1.15×10^3^	0.9990	0.005
145	*p*,*p*'-DDD (*p*,*p*'-滴滴滴)	16.300	235.00>165.00, 237.00>165.00	24, 28	*y*=1.66×10^6^*x*-6.73×10^3^	0.9999	0.005
146	fenthionsulfone (倍硫磷砜)	16.325	310.00>105.20, 310.00>109.00	12, 21	*y*=8.91×10^4^*x*-3.84×10^2^	0.9992	0.005
147	aclonifen (苯草醚)	16.334	212.00>182.10, 264.00>194.10	15, 18	*y*=1.41×10^5^*x*-6.02×10^2^	0.9993	0.005
148	*o*,*p*'-DDT (*o*,*p*'-滴滴涕)	16.358	235.00>165.00, 237.00>165.00	24, 28	*y*=1.66×10^6^*x*-6.39×10^3^	0.9990	0.005
149	ethion (乙硫磷)	16.383	153.00>97.00, 230.90>129.00	14, 24	*y*=4.40×10^5^*x*-1.91×10^3^	0.9994	0.005
150	oxadixyl (噁霜灵)	16.388	163.10>132.10, 132.10>117.10	8, 18	*y*=4.32×10^5^*x*-7.16×10^2^	0.9991	0.005
151	triazophos (三唑磷)	16.635	161.00>134.00, 161.00>106.00	8, 14	*y*=2.87×10^5^*x*-1.26×10^2^	0.9996	0.005
152	famphur (伐灭磷)	16.766	218.00>109.00, 218.00>79.00	16, 24	*y*=5.55×10^5^*x*-2.14×10^3^	0.9999	0.005
153	carbophenothion (三硫磷)	16.781	157.00>45.00, 341.90>157.00	18, 14	*y*=2.41×10^5^*x*-1.00×10^3^	0.9991	0.005
154	quinoxyfen (喹氧灵)	16.878	237.10>208.10, 307.10>237.10	28, 22	*y*=5.01×10^5^*x*-1.85×10^3^	0.9994	0.005
155	propiconazole (丙环唑)	16.910, 17.027	173.00>145.00, 259.00>69.00	16, 14	*y*=4.09×10^5^*x*+6.35×10^3^	0.9994	0.005
156	trifloxystrobin (肟菌酯)	16.964	116.00>89.00, 131.00>89.00	15, 30	*y*=5.15×10^5^*x*-8.16×10^2^	0.9991	0.005
157	*p*,*p*'-DDT (*p*,*p*'-滴滴涕)	16.984	235.00>165.00, 237.00>165.00	24, 28	*y*=5.53×10^5^*x*-2.40×10^3^	0.9993	0.005
158	hexazinone (环嗪酮)	17.195	171.10>71.00, 171.10>85.00	16, 16	*y*=8.47×10^5^*x*-3.50×10^3^	0.9992	0.005
159	tebuconazole (戊唑醇)	17.246	250.10>125.10, 125.10>89.00	22, 18	*y*=2.61×10^5^*x*-1.01×10^3^	0.9998	0.005
160	diclofop (禾草灵)	17.271	340.00>253.00, 253.00>162.00	14, 22	*y*=2.19×10^5^*x*-3.29×10^2^	0.9998	0.005
161	piperonylbutoxide (增效醚)	17.381	176.10>131.10, 176.10>117.10	12, 20	*y*=5.74×10^5^*x*-1.61×10^3^	0.9992	0.005
162	epoxiconazole (氟环唑)	17.564	192.00>138.00, 192.00>111.00	14, 26	*y*=5.52×10^5^*x*-1.81×10^3^	0.9991	0.005
163	tetramethrin (胺菊酯)	17.818, 17.937	164.10>107.10, 164.10>77.00	14, 22	*y*=7.04×10^5^*x*-1.80×10^3^	0.9997	0.005
164	pyridaphenthion (哒嗪硫磷)	17.816	340.00>199.10, 199.10>92.00	8, 16	*y*=2.09×10^5^*x*-9.46×10^2^	0.9994	0.005
165	phosmet (亚胺硫磷)	17.901	160.00>77.00, 160.00>133.00	24, 14	*y*=6.19×10^5^*x*-2.49×10^3^	0.9997	0.005
166	bromopropylate (溴螨酯)	17.906	340.90>182.90, 340.90>184.90	18, 20	*y*=4.23×10^5^*x*-1.22×10^3^	0.9991	0.005
167	bifenthrin (联苯菊酯)	17.933	181.10>166.10, 181.10>179.10	12, 12	*y*=1.76×10^6^*x*-5.68×10^2^	0.9992	0.005
168	EPN (苯硫磷)	17.932	156.90>77.00, 169.10>77.00	24, 22	*y*=3.50×10^5^*x*-9.56×10^2^	0.9995	0.005
169	methoxychlor (甲氧滴滴涕)	18.027	227.10>169.10, 227.10>212.10	24, 14	*y*=4.22×10^5^*x*-1.58×10^2^	0.9987	0.005
170	fenpropathrin (甲氰菊酯)	18.058	181.10>152.10, 265.10>210.10	22, 12	*y*=2.44×10^5^*x*-9.75×10^2^	0.9998	0.005
171	dicofol (三氯杀螨醇)	18.102	139.00>111.00, 139.00>75.00	16, 28	*y*=2.29×10^5^*x*-3.78×10^2^	0.9996	0.005
172	tebufenpyrad (吡螨胺)	18.122	333.10>171.10, 333.10>276.10	20, 8	*y*=2.77×10^5^*x*-4.99×10^2^	0.9990	0.005
173	fenamidone (咪唑菌酮)	18.162	238.00>237.20, 268.10>180.10	10, 16	*y*=5.69×10^5^*x*-1.51×10^3^	0.9992	0.005
174	bifenox (氟萘禾草灵)	18.238	340.90>309.90, 340.90>188.90	10, 20	*y*=8.34×10^4^*x*-3.67×10^2^	0.9991	0.005
175	anilofos (莎稗磷)	18.273	226.10>157.00, 226.10>184.00	14, 6	*y*=3.54×10^5^*x*-1.10×10^3^	0.9998	0.005
176	tetradifon (三氯杀螨砜)	18.410	226.90>199.00, 355.90>159.00	16, 18	*y*=1.02×10^5^*x*-2.23×10^2^	0.9994	0.005
177	pyriproxyfen (吡丙醚)	18.658	136.10>78.00, 136.10>96.00	20, 14	*y*=4.55×10^5^*x*+3.42×10^3^	0.9995	0.005
178	leptophos (溴苯磷)	18.623	376.90>361.90, 374.90>359.90	24, 24	*y*=1.64×10^5^*x*-4.00×10^2^	0.9995	0.005
179	mefenacet (苯噻酰草胺)	18.841	192.00>136.00, 192.00>109.00	14, 24	*y*=9.36×10^5^*x*-2.60×10^3^	0.9991	0.005
180	iambda (高效氯氟氰菊酯)	18.923	208.00>181.00, 197.00>141.00	8, 12	*y*=5.37×10^5^*x*-2.06×10^3^	0.9999	0.005
181	acrinathrin (氟丙菊酯)	19.094	289.10>93.00, 289.10>77.00	14, 26	*y*=9.61×10^4^*x*-3.49×10^2^	0.9994	0.005
182	fenarimol (氯苯嘧啶醇)	19.091	251.00>139.00, 330.00>139.00	14, 8	*y*=1.88×10^5^*x*-6.62×10^2^	0.9993	0.005
183	pyrazophos (定菌磷)	19.172	221.10>193.10, 221.10>149.10	12, 14	*y*=4.42×10^5^*x*-1.51×10^3^	0.9993	0.005
184	azinphos (乙基谷硫磷)	19.241	160.10>132.10, 132.10>77.00	4, 14	*y*=5.58×10^5^*x*-2.02×10^3^	0.9997	0.005
185	permethrin (氯菊酯)	19.645, 19.774	183.10>153.10, 183.10>168.10	14, 14	*y*=3.89×10^5^*x*-8.18×10^2^	0.9992	0.005
186	pyridaben (哒螨灵)	19.795	147.10>117.10, 147.10>132.10	22, 14	*y*=1.34×10^6^*x*-5.23×10^3^	0.9991	0.005
187	fluquinconazole (氟喹唑)	19.891	340.00>298.00, 340.00>313.00	20, 14	*y*=2.55×10^5^*x*-7.39×10^2^	0.9995	0.005
188	coumaphos (蝇毒磷)	19.923	362.00>109.00, 362.00>226.00	16, 14	*y*=1.25×10^5^*x*-5.17×10^2^	0.9998	0.005
189	cyfluthrin (氟氯氰菊酯)	20.236, 20.324,	226.10>206.10, 198.90>170.10	14, 25	*y*=1.77×10^5^*x*-7.54×10^2^	0.9998	0.005
		20.405, 20.427					
190	cypermethrin (氯氰菊酯)	20.548, 20.640,	163.10>127.10, 163.10>91.00	6, 14	*y*=3.77×10^5^*x*-1.28×10^3^	0.9998	0.005
		20.718, 20.749					
191	boscalid (啶酰菌胺)	20.659	140.10>112.10, 140.10>76.00	12, 24	*y*=1.09×10^6^*x*-4.05×10^3^	0.9998	0.005
192	flucythrinate (氟氰戊菊酯)	20.762, 20.950	199.10>157.10, 157.10>107.10	10, 12	*y*=8.19×10^5^*x*-2.27×10^3^	0.9991	0.005
193	fenvalerate (氰戊菊酯)	21.462, 21.665	225.10>119.10, 225.10>147.10	20, 10	*y*=1.59×10^5^*x*-5.34×10^2^	0.9998	0.005
194	fluvalinate (氟胺氰菊酯)	21.642, 21.695	250.10>55.00, 250.10>200.00	20, 10	*y*=3.25×10^5^*x*-1.24×10^3^	0.9996	0.005
195	difenoconazole (苯醚甲环唑)	21.930, 21.990	323.00>265.00, 265.00>202.00	14, 20	*y*=5.10×10^5^*x*-1.76×10^3^	0.9991	0.005
196	deltamethrin (溴氰菊酯)	22.021, 22.239	180.90>151.90, 252.90>93.00	22, 20	*y*=1.23×10^5^*x*-3.48×10^2^	0.9975	0.005

BHC: hexachlorocyclohexane; *y*: peak area; *x*: mass concentration, mg/L.

### 1.2 样品前处理

#### 1.2.1 样品提取

准确称取5 g(精确到0.01 g)匀浆样品至50 mL具塞离心管中,加入10 mL乙腈,涡旋提取5 min,加入4 g氯化钠,涡旋混匀1 min, 8500 r/min离心3 min,取上清液5 mL,待净化。

#### 1.2.2 样品净化

将5 mL上清液移取至QuEChERS净化管(900 mg MgSO_4_+150 mg PSA+150 mg C18)中,涡旋2 min, 5000 r/min离心2 min后,取2 mL上清液至15 mL离心管中,于40 ℃水浴中氮吹至近干,加入1 mL乙酸乙酯复溶残渣,溶液过0.22 μm有机相滤膜后,经在线GPC-GC-MS/MS测定。

### 1.3 实验条件

#### 1.3.1 凝胶渗透色谱条件

凝胶色谱柱:Shodex CLNpak EV-200AC(150 mm×2 mm);紫外检测波长254 nm;流动相为丙酮-环己烷(3∶7, v/v)混合溶液,流速为0.1 mL/min;柱温40 ℃;进样量10 μL;开始收集时间3.49 min,结束收集时间5.50 min。

#### 1.3.2 凝胶渗透色谱-气相色谱接口

样品溶液在GPC中进行分离,收集所需时间段内的馏分并自动导入至气相色谱进样口中,开始时进样口中的温度较低,目标物不能进行汽化,待目标物全部导入后,进样口温度快速升温,目标物进入色谱柱中进行分离。进样口升温程序:起始温度为120 ℃,保持5 min,以100 ℃/min升温至300 ℃,保持19.7 min。

#### 1.3.3 色谱条件

色谱柱:预柱为DB-5MS石英毛细管柱(5 m×0.25 mm×0.25 μm,美国Agilent公司),分析柱为DB-5MS(25 m×0.25 mm×0.25 μm,美国Agilent公司);色谱柱升温程序:起始温度为50 ℃,保持1 min,以25 ℃/min程序升温至125 ℃,再以10 ℃/min程序升温至300 ℃,保持5 min;载气为氦气,纯度≥99.999%。

#### 1.3.4 质谱条件

离子源:电子轰击源(EI);一级质谱电离能:70 eV;离子源温度:200 ℃;传输线温度:300 ℃;溶剂延迟:5 min;采集数据模式:多反应监测(MRM)模式,监测离子对及碰撞能量见表1。

## 2 结果与讨论

### 2.1 GC-MS/MS条件的优化

将196种农药混合标准溶液进行GC-MS/MS全扫描,与NIST谱库进行比对,得出每种农药的保留时间和离子碎片,选取相对丰度较大的离子作为前级离子,使用不同碰撞能进行碰撞,得到各化合物的二级碎片离子及最优碰撞能量,相关数据见表1。

### 2.2 提取溶剂的优化

本实验目标物种类较多,选择提取溶剂时要同时考虑样品基质的复杂性和目标物的物理化学性质。据文献报道,农药残留检测常用的提取溶剂有乙腈、丙酮和乙酸乙酯等,本文依据文献对这3种提取溶剂进行了优化。结果显示,乙酸乙酯作为提取溶剂时,其提取液经净化后进行氮吹,吹至近干时有胶状物出现,尤其是猪肉样品,氮吹至近干后胶状物质含量较高,复溶时无法均匀分散。这可能是因为乙酸乙酯是一种中等极性的有机溶剂,其能将样品中弱极性的脂肪、极性或含有离子基团的磷脂提取出来,而这些脂肪和磷脂影响实验的进行,故乙酸乙酯不适合作为提取溶剂。乙腈和丙酮作为提取溶剂时,196种农药的回收率分别为71.5%~118.6%和73.4%~116.9%,均可满足检验要求。

将乙腈提取液和丙酮提取液分别进行GC-MS/MS全扫描,结果发现,丙酮作为提取溶剂时,杂质峰比较多,可能是由于采用丙酮提取时溶入的油脂量大,提取出的杂质较多;乙腈作为提取溶剂时,全扫描色谱图相对比较干净,且大多数农药具有更好的色谱分辨率和更高的灵敏度。采用丙酮提取时共萃取物较多,导致部分农药的色谱峰形较差,出现色谱峰分叉的现象,而乙腈具有较强的沉淀蛋白质作用^[23]^,将其作为提取剂时可在一定程度上减少基质干扰,莠灭净、扑草净、去草净、乙嘧酚磺酸酯的色谱峰形得到明显改善,峰形尖锐且对称,如图1所示,故实验选用乙腈作为提取溶剂。

**图1:**
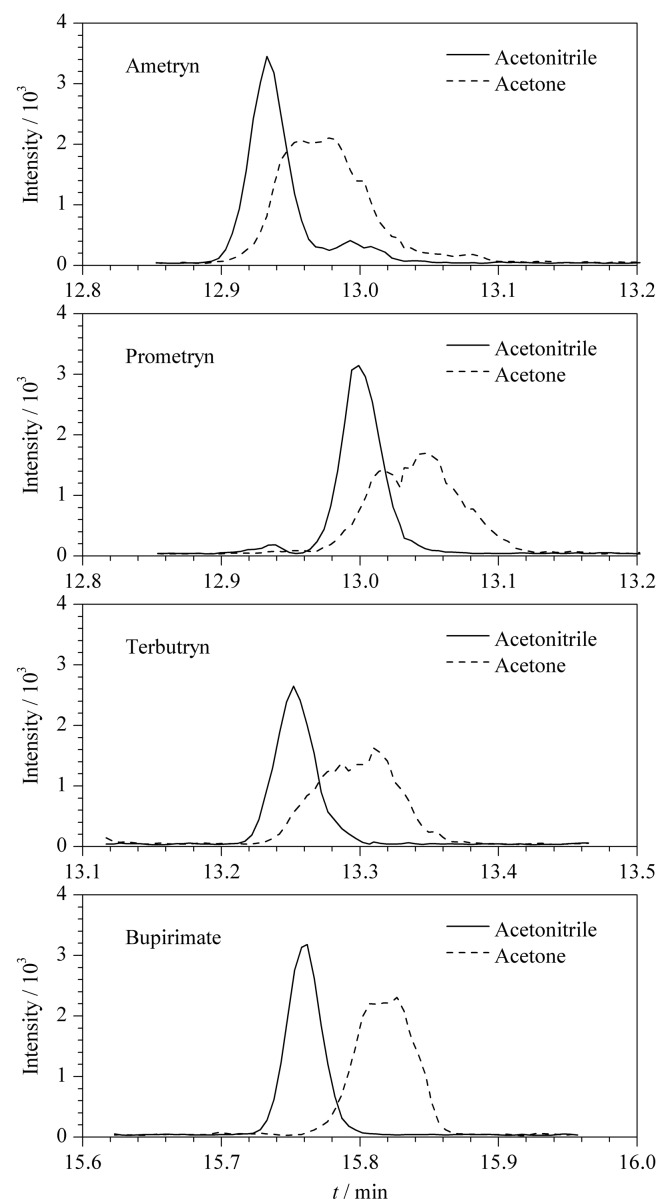
乙腈和丙酮作为提取溶剂时4种农药的选择离子色谱图

### 2.3 净化条件的优化

动物源性食品基质复杂,含有小分子的有机酸和大分子的脂肪、蛋白质等杂质,单一的净化手段难以同时去除这两类杂质,不能满足实验要求。农药常用的净化方式有QuEChERS净化、固相萃取净化、分散固相萃取净化等,主要去除的是小分子有机酸和少量的大分子杂质,而大部分的大分子杂质仍未去除。GPC可将大分子化合物有效去除,将两种净化方式结合,可最大限度地减少共萃取物的残留。

#### 2.3.1 净化方式的选择

实验考察了QuEChERS(900 mg MgSO_4_+150 mg PSA+150 mg C18)、EMR-lipid净化剂、C18固相萃取柱(1 g/6 mL)、氨基柱(500 mg/6 mL)、弗罗里硅土柱(1 g/6 mL)、HLB固相萃取柱(60 mg/3 mL)6种净化方式对样品提取液的净化效果,6种净化方式的具体操作步骤见附表1(www.chrom-China.com)。将6种净化液进行GC-MS/MS全扫描,其色谱图见图2a。HLB固相萃取柱对目标物的保留性强,利用极性较强的乙腈-甲苯混合溶液可将目标物有效洗脱下来,但同时某些极性杂质也会被共洗脱下来,增加了基质干扰;Florisil柱是硅胶键合氧化镁制得的吸附剂,其与硅胶相似,是强极性吸附剂,仅可吸附萃取某些极性化合物杂质;EMR-lipid净化剂对脂类基质的吸附效果良好,但对于极性基质的去除效果不明显;PSA吸附剂和氨基柱均具有极性固定相和弱阴离子交换剂,可通过弱阴离子交换作用和极性相互作用吸附色素和脂肪酸等,而对于弱极性或非极性杂质的净化效果差;C18材料是一种疏水性吸附剂,对非极性的组分和弱极性的干扰物如脂肪酸、氨基酸、脂肪和脂类等有较好的吸附效果^[24⇓-26]^。因此将PSA与C18结合可覆盖较宽的极性范围,有效去除肉类基质中大部分的杂质,由图2a也可以看出,QuEChERS作为净化剂时,色谱峰基线最平稳,杂质峰最少。

**图2:**
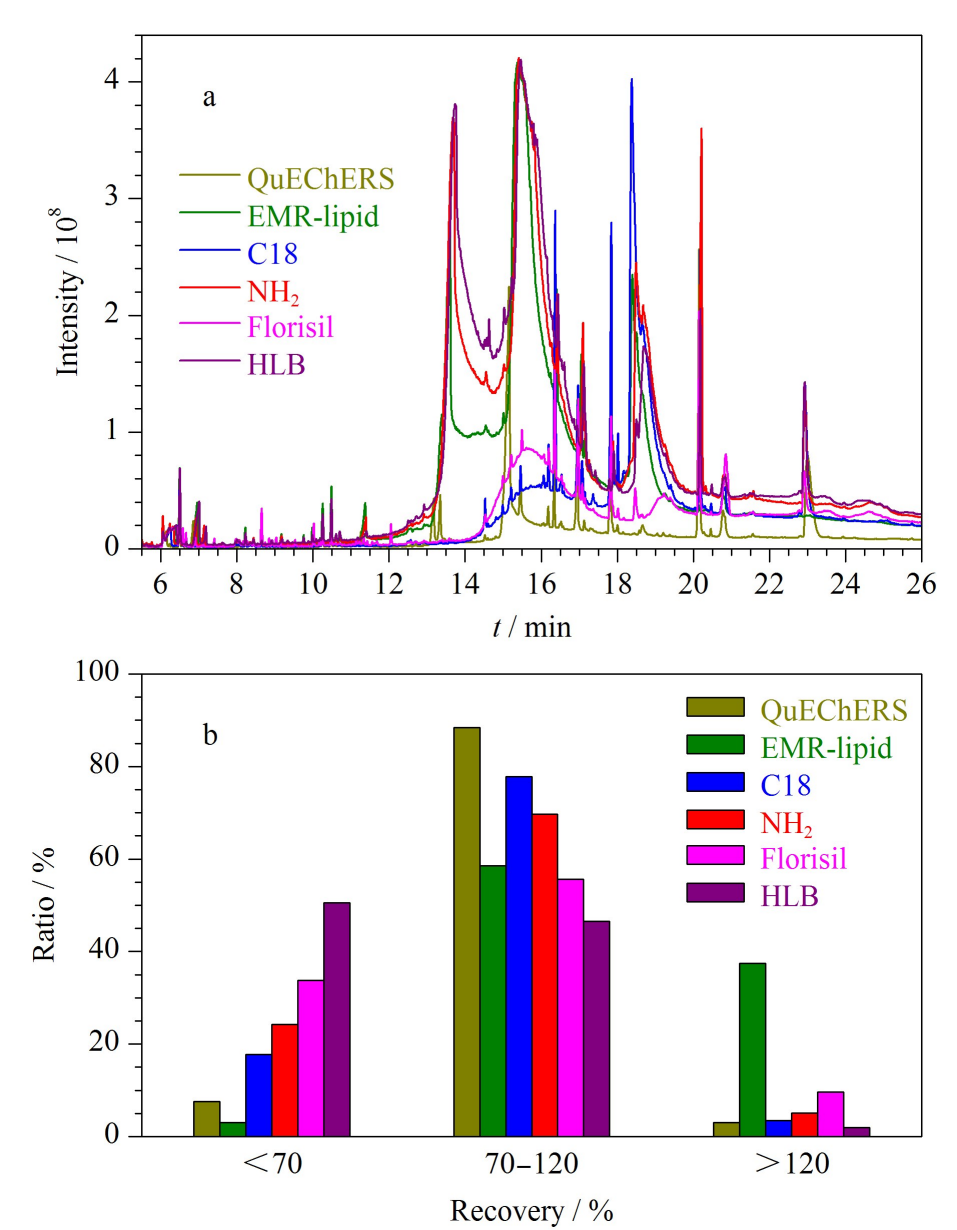
(a)6种净化液的色谱图和(b)不同净化方式下196种农药加标回收率的分布比例

本实验同时考察了6种净化方式对196种农药加标回收率的影响。在空白样品中添加含量为20 μg/kg的混合标准溶液,比较6种净化方式的回收率,结果见图2b。实验发现QuEChERS作为净化剂时,回收率为70%~120%的农药最多,占比为88.4%,说明二者对肉类基质具有较好的净化性能;HLB固相萃取柱和Florisil固相萃取柱作为吸附剂时,对极性目标物的保留性强,导致某些有机磷回收率较差,回收率低于70%的农药占比分别高达50.5%和33.8%;EMR-lipid净化剂由于净化效果差,增加了农药的基质效应(ME),回收率大于120%的农药占比达到了37.4%。综合考虑净化剂对基质的去除效果和目标物的回收率,选用QuEChERS作为最终的净化方式。

#### 2.3.2 GPC条件的优化

本研究选用目标物中相对分子质量最大的溴氰菊酯(505.2)和相对分子质量最小的嘧霉胺(199.25)作为参照物,将溴氰菊酯标准溶液(质量浓度为10 μg/mL)、嘧霉胺标准溶液(质量浓度为10 μg/mL)和空白基质溶液分别注入在线GPC中,其凝胶渗透色谱图见图3a。空白基质溶液、溴氰菊酯和嘧霉胺标准品的保留时间分别为3.26、3.77和4.18 min,从图3a中可以看到,收集不同时间段内的馏分,可实现样品中大分子杂质和目标物的有效分离。

**图3:**
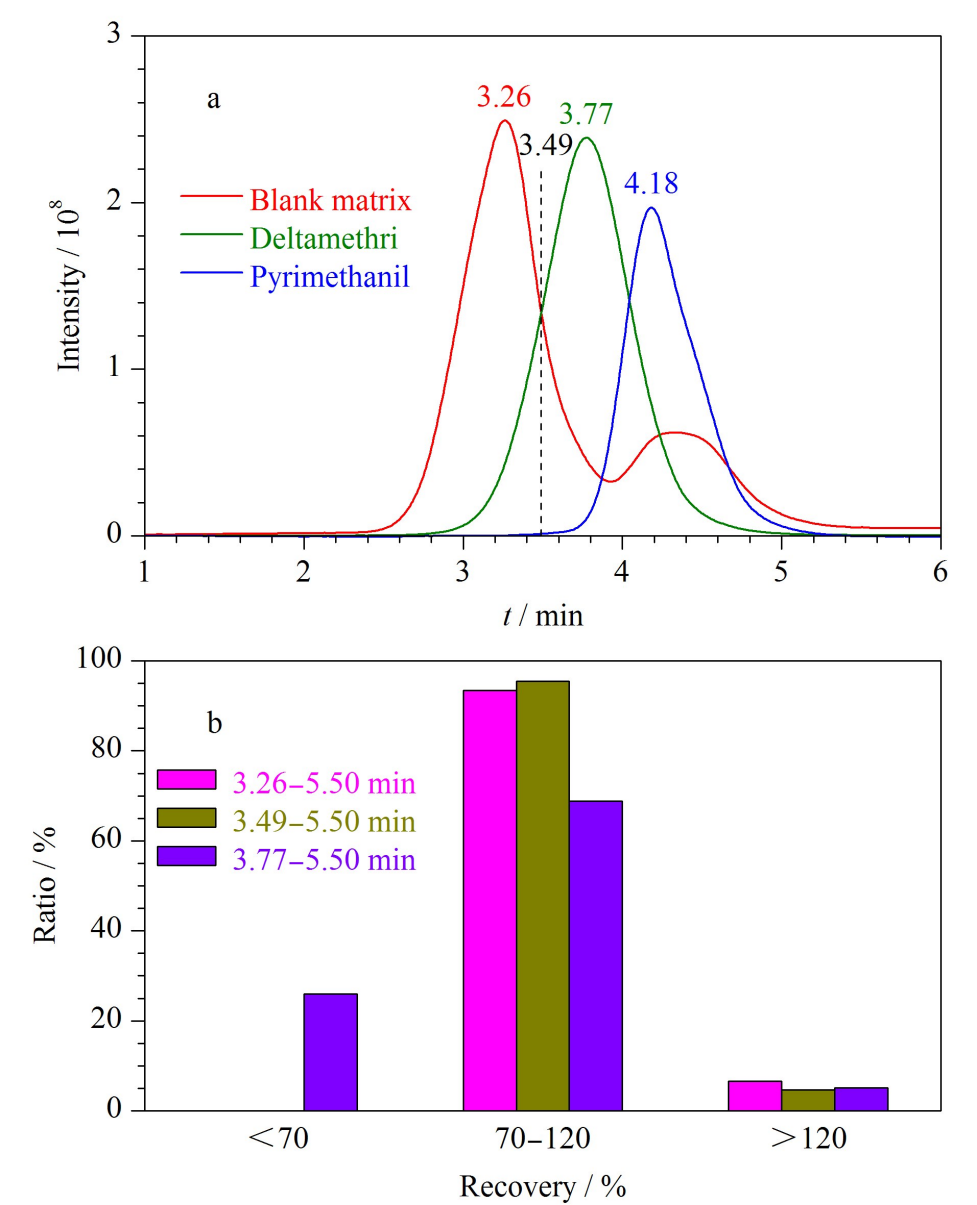
(a)空白基质溶液、溴氰菊酯和嘧霉胺标准溶液的凝胶渗透色谱图和(b)不同馏分中196种农药的加标回收率分布比例

由于目标物种类多,涵盖的相对分子质量范围较宽,且样品基质复杂,少部分杂质和目标物的流出时间重叠,为既能保证目标物的回收率,又能去除杂质,本实验对接收馏分的时间段进行了优化。由图3a可知,在3.77 min时接收流出组分可将大部分大分子杂质去除,但可能会有部分目标物随非接收组分流出,造成回收率降低;在3.26 min时接收流出组分,目标物可全部转移至气相色谱中,但此馏分中的杂质含量也较高,可能影响净化效果。

本实验分别考察了接收3.26~5.50 min、3.49~5.50 min和3.77~5.50 min 3个时间段馏分时196种农药的回收率,结果见3b。结果显示,3.77~5.50 min时间段内农药回收率低于70%的有51种,尤其对于相对分子质量较大的氟氯氰菊酯、氯氰菊酯、氟氰戊菊酯、氰戊菊酯、氟胺氰菊酯、溴氰菊酯等化合物,回收率仅为19.1%~58.2%,这是因为相对分子质量较大的化合物随大分子杂质一起排入了废液中,影响检测的灵敏度和准确性。3.26~5.50 min时间段和3.49~5.50 min时间段内的农药回收率均大于70%,满足检测要求。由图3a所示,3.26~5.50 min时间段内的馏分中仍有部分大分子杂质跟随目标物进入色谱柱中,吸附在进样口和柱头,对目标物的峰形产生负面影响,影响目标物的定性和定量;并且会对气相色谱柱造成污染,使色谱柱分离效果下降,缩短色谱柱使用寿命并干扰色谱测定。综合考虑农药的回收率和基质的去除效果,最终选择3.49~5.50 min时间段内的馏分作为接收馏分。

#### 2.3.3 QuEChERS-GPC联用技术的优势

利用优化的提取溶剂对样品进行处理后,分别采用QuEChERS、在线GPC、QuEChERS+在线GPC 3种方式对样品溶液进行净化后,进行GC-MS/MS分析,比较了3种净化方式的净化效果,其扫描色谱图见图4。通过对比发现,仅用在线GPC净化的样品溶液在色谱图中的b、c时间段内均有明显的杂质峰;只经过QuEChERS净化的样品溶液在b、c时间段内的色谱峰响应相对较低,但在a时间段内有群峰出现;经过二者双重净化后,在a、b、c 3个时间段内的色谱峰数量明显减少,且强度均有所下降,基线较平滑。通过NIST谱库检索,a时间段内的杂质峰主要为一些小分子化合物,GPC对不同相对分子质量的化合物具有较好的分离能力,因此该时间段内样品溶液经GPC净化后杂质峰较少;b、c时间段内的杂质峰主要为棕榈酸、油酸及其甘油酯、胆固醇,PSA和C18对有机酸和非极性化合物有较好的吸附作用,故经QuEChERS净化后,此时间段内的杂质峰得到了高效去除。由于QuEChERS仅通过极性大小进行吸附,对于与农药极性相近的化合物,无法将其全部去除,而GPC柱能去除特定分子大小的化合物,可以弥补QuEChERS的缺点,因此在该实验中QuEChERS和在线GPC两种净化方式缺一不可。

**图4:**
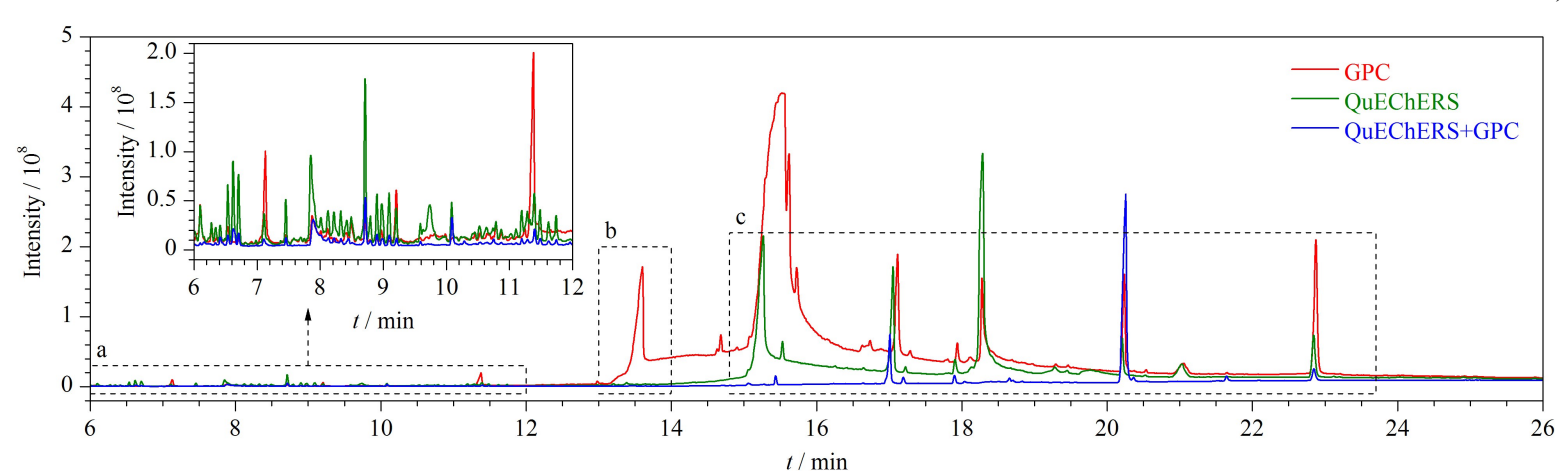
不同净化方式下样品溶液的全扫描色谱图

### 2.4 基质效应

样品分析溶液在注入仪器后,由于共洗脱物的存在,目标物的信号可能会增强或减弱,进而产生基质效应。本文通过测定溶剂标准曲线和基质匹配标准曲线的斜率来评价基质效应,ME=基质匹配标准曲线斜率/溶剂标准曲线斜率。利用脂肪含量较高的空白猪肉样品进行基质效应的考察,将空白猪肉样品按1.2节的实验步骤进行前处理,再将经QuEChERS净化后的提取液配制成空白基质标准溶液;分别将溶剂标准溶液和空白基质标准溶液进行GC-MS/MS和在线GPC-GC-MS/MS分析,考察经过在线GPC净化前后196种农药的基质效应,结果如图5所示。结果显示,未经在线GPC净化,196种农药中有143种农药表现为基质增强效应,其中35种农药的ME为1.20~1.50,表现为中等基质增强效应,12种农药的ME>1.50,表现为强基质增强效应,严重影响定量,分析其原因可能是由于净化不彻底,基质对目标物产生了正干扰;而经在线GPC净化后,仅有10种农药的ME为1.20~1.50,表现出中等基质增强效应,4种农药的ME>1.50,表现出强基质增强效应,进一步说明了在线GPC可有效去除影响定量的基质干扰。肉类品种较多,基质较为复杂,为满足不同肉类的分析要求、降低基质效应带来的影响,本实验采用基质匹配标准溶液进行定量分析。

**图5:**
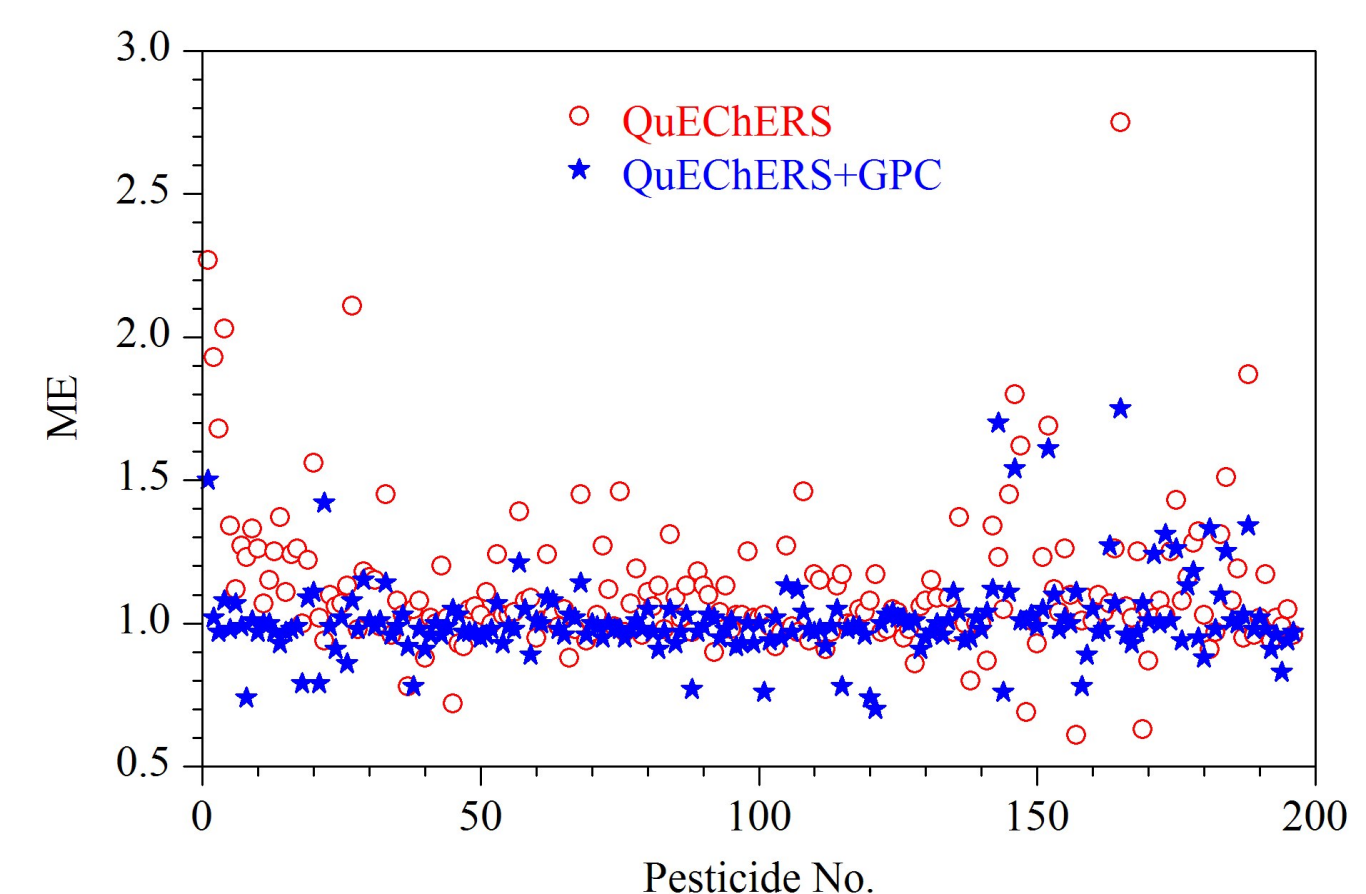
196种农药的基质效应

### 2.5 方法学评价

#### 2.5.1 线性范围、相关系数及定量限

用猪肉样品空白基质配制196种农药的系列混合标准溶液,质量浓度为0.005、0.01、0.02、0.05、0.1、0.2 mg/L,以定量离子的峰面积对相应的质量浓度绘制标准曲线。结果表明,196种农药在0.005~0.2 mg/L范围内具有良好的线性关系,且相关系数均大于0.996,相关数据见表1。向猪肉样品空白基质样品中添加不同水平的目标化合物,以信噪比(*S/N*)≥3时对应的添加水平为检出限(LOD), *S/N*≥10时对应的添加水平为定量限(LOQ),结果显示,196种农药的检出限为0.002 mg/kg,定量限为0.005 mg/kg,相关化合物的检出限可满足GB 2763-2021《食品安全国家标准 食品中农药最大残留限量》和其他国家组织对动物源性食品中农药残留的限量要求。

#### 2.5.2 回收率和精密度

向猪肉、牛肉、猪肝3种不同脂肪含量基质的空白样品中添加标准溶液,利用本研究建立的检测方法在3个加标水平下(0.01、0.05、0.20 mg/kg)进行加标回收试验,每个加标水平重复6次,结果见附表2。测得方法的平均回收率为65.3%~126.2%,精密度为0.7%~5.7%,符合农药残留检测的要求。

### 2.6 实际样品的检测

利用建立的方法,分别对市售的8批猪肉、6批牛肉、4批羊肉、6批鸡肉、3批猪肝、1批猪肾、1批猪肺和1批牛肝等30批次样品进行了检测。结果显示,30批样品中有1批次牛肉检出氯菊酯,含量为0.015 mg/kg,检出值低于中国、欧盟、日本、美国等国家的MRL规定;1批次牛肉检出胺菊酯,含量为0.004 mg/kg(我国GB 2763-2021并未对此农药作限量要求),其他农药均未检出。

## 3 结论

本方法建立了GC-MS/MS测定动物源性食品中196种农药残留的定性、定量分析方法。采用乙腈提取,可实现目标物的高效提取和蛋白质的沉淀,使得目标物保留良好、色谱峰形对称。利用QuEChERS结合在线GPC净化技术,可有效去除动物源性食品中的大、小分子杂质。在线GPC的应用降低了溶剂的消耗量,缩短了分析时间。利用GC-MS/MS对目标物进行高通量检测,可实现农药残留的精准定性和精确定量,结果准确、可靠,灵敏度高,能够满足动物源性食品中多种农药残留检测的需求,可为多农药残留的监测提供技术支持。

## References

[1] Dai W, Li Q, Zhu M, et al. Chinese Journal of Chromatography, 2021, 39(11): 1213 34677016 10.3724/SP.J.1123.2021.01029 PMC9404141

[2] Li G Q, Wang X L. Chemical Reagents, 2019, 41(9): 958

[3] GB 2763-2005

[4] GB 2763-2021

[5] Lian Y J, Zhou Y R, Sun X, et al. Food Science, 2021, 42(4): 297

[6] Liu Z R, Zhang M T, Xie N, et al. Journal of Chinese Mass Spectrometry Society, 2020, 41(6): 624

[7] Yang Z M, Zhu R Y, Xue H L, et al. Chinese Journal of Analysis Laboratory, 2023, 42(1): 49

[8] González D M, Durán J A, Silvina M A, et al. J Chromatogr A, 2018, 1562: 27 29861303

[9] Wang S W, Sun H B, Liu Y P, et al. Food Science, 2021, 42(20): 310

[10] Xie M, Zhao L J, Zhou Y M, et al. Chinese Journal of Analysis Laboratory, 2023, 42(2): 203

[11] Xu R H, Xie Q W, Li X J, et al. Chinese Journal of Chromatography, 2022, 40(5): 469 35478006 10.3724/SP.J.1123.2021.11015 PMC9404200

[12] Liu S N, Tian X, Gao J, et al. Food Science, 2022, 43(4): 284

[13] Zhang Q, Bi S, Wu Y T, et al. Chinese Journal of Chromatography, 2022, 40(6): 565 35616202 10.3724/SP.J.1123.2021.12010 PMC9404037

[14] Pan S D, Guo Y B, Wang L, et al. Chinese Journal of Chromatography, 2021, 39(6): 614 34227322 10.3724/SP.J.1123.2020.11011 PMC9404219

[15] Lü B, Chen D W, Miao H. Journal of Instrumental Analysis, 2015, 34(6): 639

[16] GB/T 19650-2006

[17] GB/T 20772-2008

[18] Zhao K X, Pan Y X, Yang S. Physical Test and Chemical Analysis (Part B: Chemical Analysis), 2019, 55(3): 297

[19] Li M M, Shen X X, Wang S S, et al. Journal of Nanjing Agricultural University, 2022, 45(6): 1246

[20] Han B J, Huang H Z, He Y, et al. Food Research and Development, 2017, 38(20): 130

[21] Tang W L, Yi S F, Deng M, et al. Journal of Food Safety and Quality, 2020, 11(24): 9124

[22] Lü F, Li H D, Ye Y, et al. Chinese Journal of Food Hygiene, 2016, 28(1): 69

[23] Xiao Y, Deng H, Pan Z, et al. Food and Fermentation Industries, 2022, 48(10): 272

[24] Xu X, Zhang Y, Shu P, et al. Science and Technology of Food Industry, 2022, 43(24): 320

[25] Chen X L, Li Q, Wang Z F, et al. Journal of Chinese Mass Spectrometry Society, 2021, 42(6): 1046

[26] Hakme E, Lozano A, Ferrer C, et al. TrAC-Trends Anal Chem, 2018, 100: 167

